# miR-335-5p suppresses gastric cancer progression by targeting MAPK10

**DOI:** 10.1186/s12935-020-01684-z

**Published:** 2021-01-22

**Authors:** Yi Gao, Yanfeng Wang, Xiaofei Wang, Changan Zhao, Fenghui Wang, Juan Du, Huahua Zhang, Haiyan Shi, Yun Feng, Dan Li, Jing Yan, Yan Yao, Weihong Hu, Ruxin Ding, Mengjie Zhang, Lumin Wang, Chen Huang, Jing Zhang

**Affiliations:** 1 Department of Cell Biology and Genetics, Medical College of Yan’an University, Yan’an, 716000 Shaanxi China; 2 Yan’an Key Laboratory of Chronic Disease Prevention and Research, Yan’an, 716000 Shaanxi China; 3 Department of Medical Genetic and Cell Biology, Ningxia Medical University, Yinchuan, 750004 China; 4 Department of Cell Biology and Genetics, School of Basic Medical Sciences, Xi’an Jiaotong University Health Science Center, Xi’an, 710061 Shaanxi China; 5 Department of Pathology, School of Basic Medical Sciences, Xi’an Jiaotong University, Xi’an, China

**Keywords:** miR-335-5p, Gastric cancer, MAPK10, Proliferation

## Abstract

**Background:**

Recent studies have established the roles of microRNAs (miRNAs) in cancer progression. The aberrant expression of miR-335-5p has been reported in many cancers, including gastric cancer (GC). In this study, the precise roles of miR-335-5p in GC as well as the molecular mechanisms underlying its effects, including the role of its target MAPK10, were evaluated.

**Methods:**

Quantitative real-time PCR was used to evaluate miR-335-5p levels in GC cell lines and tissues. MTT and colony formation assays were used to detect cell proliferation, and Transwell and wound-healing assays were used to evaluate the invasion and migration of GC cells. The correlation between levels of miR-335-5p and the cell cycle-related target gene mitogen-activated protein kinase 10 (MAPK10) in GC was analyzed. In addition, the candidate target was evaluated by a luciferase reporter assay, qRT-PCR, and western blotting.

**Results:**

The levels of miR-335-5p were downregulated in GC tissues and cell lines. Furthermore, miR-335-5p inhibited the proliferation and migration of GC cells and induced apoptosis. Additionally, miR-335-5p arrested the cell cycle at the G1/S phase in GC cells in vitro. Levels of miR-335-5p and the cell cycle-related target gene MAPK10 in GC were correlated, and MAPK10 was directly targeted by miR-335-5p.

**Conclusions:**

These data suggest that miR-335-5p is a tumor suppressor and acts via MAPK10 to inhibit GC progression.

## Background

Gastric cancer (GC) is still a significant public health problem worldwide [1, 2]. Over 1,000,000 new cases and an estimated 783,000 deaths were reported in 2018, making it the fifth most frequently diagnosed cancer and the third leading cause of cancer deaths [3]. A wide range of factors, such as lifestyle, Helicobacter pylori infection, polyps, gastric ulcers, genetic factors, and gastric residual tissue, may be involved in gastric tumorigenesis [4]. Although there are many methods for the diagnosis and treatment of GC, 30% of patients are diagnosed at an advanced stage [5]. Thus, useful biomarkers for early screening or detection are essential for improving survival rates [6].

Recent studies have established the important roles of microRNAs (miRNAs), which are small, non-protein-coding RNAs that regulate gene expression at the post-transcriptional level, in tumorigenesis [7–9]. Mature miRNAs of about 22 nucleotides contribute to gastric carcinogenesis by altering the expression of oncogenes and tumor suppressors [10]. For example, miR-181d [11], miR-99a [12], miR-105 [13], and others have suppressive effects on GC development, whereas other miRNAs, including miR-188-5p [14] and miR-221 [15], promote GC growth.

MiR-335-5p is abnormally expressed in many cancers. For instance, miR-335-5p is significantly downregulated and has a vital role in the metastasis of non-small cell lung cancer [16]. miR-335-5p is downregulated in breast cancer cells and is a promising biomarker for breast cancer treatment [17]. Additionally, miR-335-5p is downregulated in renal cell carcinoma and is a candidate therapeutic target [18]. The downregulation of miR-335 in GC has been reported [19]; however, its precise roles in GC cells are not fully understood.

MiRNAs function by binding to the 3′UTR of target mRNAs in a complementary base-pairing manner, thereby contributing to cell apoptosis, proliferation, and differentiation [20–23]. They are thought to regulate more than 50% of protein-coding genes. Based on a literature review and gene target prediction algorithms, including TargetScan, miRanda, and miRBase, we hypothesized that mitogen-activated protein kinase 10 (MAPK10) is a potential target of miR-335-5p. MAPK10 is a member of the Jun N-terminal kinase subgroup of mitogen-activated protein kinases. MAP kinases act as integration points for multiple biochemical signals and are involved in a wide variety of cellular processes, such as proliferation, differentiation, transcription regulation, and development [24, 25]. Expression patterns of MAP kinases differ depending on the tumor type. Zhang et al. showed that MAPK10 is expressed at low levels in cervical cancer tissues and cells [26]. However, the percentage of MAPK10 protein-positive cells is significantly higher in ovarian serous, mucinous, and clear cell carcinomas than in normal tissues [27].

In this study, the effect of miR-335-5p on GC progression via MAPK10 was evaluated. In particular, we compared the expression levels of miR-335 in GC tissues and cells with those in matched normal tissues and MKN-28 and SGC-7901 cell lines. In addition, we used bioinformatics approaches, luciferase assays, qRT-PCR, and western blotting to evaluate its relationship with MAPK10 and further evaluated the functions of miR-335 and MAPK10 in GC cell proliferation, metastasis, and apoptosis. Overall, our results demonstrated that miR-335 suppresses the progression of GCs by targeting MAPK10.

## Materials and methods

### Cell lines and cell culture

Human GC cell lines (AGS, BGC-823, MKN-45, MKN-28, and SGC-7901), a normal gastric epithelial cell line (GES-1), and model cells (HEK-293) were provided by the Biomedical Experiment Center of Xi’an Jiaotong University (China). The use of these cell lines was approved by the Ethics Committee of Yan’an University College of Medicine (China). Human GC cells were cultured in DMEM (PAA Laboratories, Pasching, Australia) containing 10% fetal bovine serum and RPMI1 640 medium (PAA Laboratories) at 37 °C in a 5% CO_2_ incubator. The culture medium was changed once every 2–3 days. MKN-28 and SGC-7901 cells in the logarithmic growth phase were collected and subjected to the following experiments.

### Cell transfection

GC cells in the logarithmic growth phase were digested and inoculated onto a 6-well culture plate. After cells reached 60–80% confluence, the miR-335-5p-mimics and miR-335-5p-inhibitor (GenePharma, Shanghai, China) were added to the corresponding wells for further culture for 24–48 h.

### Quantitative real-time PCR

RNA was extracted from GC cells using TRIzol. The cDNA was obtained by reverse-transcription using commercially available kits, according to the manufacturer’s instructions. Quantitative real-time PCR was performed using the PrimeScript™ RT Reagent Kit (Takara Bio, Kusatsu, Japan) and an iQ5 Optical real-time PCR System (Bio-Rad, Hercules, CA, USA). The following primer sequences were used for amplification: RT miR-335-5p, 5ʹ-GTCGTATCCAGTGCGTGTCGTGGAGTCGGCAATTGCACTGGATACGACacatttt-3ʹ; RT U6 5ʹ-CGCTTCACGAATTTGCGTGTCAT-3ʹ; miR-335-5p, forward 5ʹ-ATCCAGTGCGTGTCGTG-3ʹ and reverse 5ʹ-TGCTTCAAGAGCAATAACGA-3ʹ; U6, forward 5ʹ-GCTTCGGCAGCACATATACTAAAAT-3ʹ and reverse 5ʹ-CGCTTCACGAATTTGCGTGTCAT-3ʹ; MAPK10 forward 5ʹ-TTCTCAGGCACGGAATGG-3 and reverse 5ʹ-TAAGTTGCCATAGTGAAGATCTGAG-3ʹ; and glyceraldehyde-3-phosphate dehydrogenase (*GAPDH*), forward 5ʹ-TGAAGGTCGGAGTCAACGGATT-3ʹ and reverse 5ʹ-CCTGGAAGATGGTGATGGGATT-3ʹ. The 20-μL PCR system consisted of 10-μL of 2× RealStar Green Power Mixture, 1 μL of forward primer (10 μM), 1 μL of reverse-primer (10 μM), 2 μL of cDNA, and 6 μL of ddH_2_O. The amplification conditions were as follows: 95 °C for 10 min; 95 °C for 15 s, 60 °C for 1 min, and 72 °C for 30 s, for a total of 40 cycles. Relative expression levels of the target genes were calculated using the 2^−ΔCt^ method. *GAPDH* was used as an internal reference.

### Western Blotting

Cells were lysed with precooled radio-immunoprecipitation assay lysis buffer supplemented with protease inhibitor (Beyotime Institute of Biotechnology, Shanghai, China) for 30 min on ice. The supernatant was collected after centrifugation at 14,000 rpm at 4 °C for 20 min. The protein concentration was determined using a bicinchoninic acid protein concentration determination kit (RTP7102; Real-Times Biotechnology Co., Ltd., Beijing, China). The samples (20 μg) were subjected to 10% sodium dodecyl sulfate-polyacrylamide gel electrophoresis and then transferred to polyvinylidene difluoride membranes. A GAPDH antibody was used as an internal reference. The membranes were washed with TBST and incubated with goat anti-mouse/rabbit antibody. Color development was performed using the chemiluminescence detection method, and images of protein bands were obtained for analyses. The luminescent signal was detected by a CCD camera, and recorded and quantified with Syngene GBox (Syngene, UK).The primary and secondary antibodies were as follows: anti-GAPDH monoclonal antibody (Cell Signaling Technology, Danvers, MA, USA; 5174S, diluted 1/1000), MAPK10 (Cell Signaling Technology; 2305S, diluted 1/1000), vimentin (Cell Signaling Technology; 5741S, diluted 1/1000), E-cadherin (ProteinTech, Rosemont, IL, USA; 20874-1-AP, diluted 1/1000), β-catenin (Cell Signaling Technology; 8480S, diluted 1/1000), CDK6 (Cell Signaling Technology; 13331S, diluted 1/1000), CDK4 (Cell Signaling Technology; 12790S, diluted 1/1000), CyclinD1 (Cell Signaling Technology; 55506S, diluted 1/1000), Bcl-2 (Cell Signaling Technology; 15071S, diluted 1/1000), Bax (ProteinTech; 50599-2-Ig, diluted 1/1000), goat anti-mouse immunoglobulin G (IgG) (ProteinTech; SA00001-1, diluted 1/3000), and goat anti-rabbit IgG (ProteinTech; SA00001-2, diluted 1/3000). Each western blotting was repeated at least three times.

### MTT assay

Cell proliferation was assessed using the MTT Kit (Sigma, St Louis, MO, USA). Cells in the logarithmic growth phase were harvested and seeded on a 96-well plate. At 24, 48, and 72 h after seeding, 10 μL of MTT was added to each well and the cells were incubated for 4 h. Each well was supplemented with 150 μL of DMSO, and the optical density (OD) was recorded at 490 nm.

### Colony formation detection

Transfected cells in the logarithmic growth phase were seeded onto a 6-well plate. After 2 weeks of culture, the cells were fixed with 4% paraformaldehyde and stained with crystal violet. Images were obtained and cells were counted.

### Flow cytometry

Transfected cells in the logarithmic growth phase were inoculated onto a 6-well plate and cultured for 1 day. Cells were fixed in 70% ethanol for 24 h and treated with propidium iodide and RNase provided with the kit. The cell cycle distribution was detected by flow cytometry.

### Dual luciferase reporter assay

HEK-293 cells were divided into the miR-335-5p and pmirGLO empty vectors, miR-335-5p and pmirGLO-MAPK10-WT (GenePharma), miR-335-5p, and pmirGLO- MAPK10-MuT (GenePharma) co-transfection groups, respectively. Untreated cells were used as controls. Wild-type and mutant MAPK10 were synthesized by GeneChem (Shanghai, China) as follows: wild-type MAPK10, up 5ʹ-cATTTAACTTCTAGTTGCTCTTGCc-3ʹ and down 5ʹ-tcgagGCAAGAGCAACTAGAAGTTAAATgagct-3ʹ; and mutant MAPK10, up 5ʹ-cATTTAACTTCTAGTTGATATCGCc-3ʹ and down 5ʹ-tcgagGCGATATCAACTAGAAGTTAAATgagct-3ʹ. Cells were inoculated onto 96-well plates and cultured for 24 h. Luciferase activity was detected using a microplate reader. *Renilla* luciferase was used as an internal reference.

### Cell invasion assay

Transwell chambers (8-μm pore size; Millipore, Billerica, MA, USA) were coated with Matrigel (15 μg/filter; BD Biosciences, Franklin Lakes, NJ, USA). Cells (2.0 × 10^4^) in serum-free medium were added to the upper chamber, and the bottom wells were filled with complete medium. The cells were allowed to cross the Matrigel-coated membrane for 48 h.

### Wound-healing assay

A wound-healing assay was performed to examine metastasis. Briefly, after cells reached 90% confluence in 12-well plates, a single scratch wound was generated with a 200-μL disposable pipette tip. The extent of wound closure was measured after 48 h.

### Statistical analysis

Results are shown as means ± SEM of at least three different experiments. The SPSS22.0 was used for statistical analyses. Bioinformatics analyses were performed using the ggstatsplot package in R. Experimental data were processed using GraphPad Prism7.0. Comparisons were conducted with the independent *t*-test. *P* < 0.05 was considered statistically significant.

## Results

### miR-335-5p inhibits GC cell proliferation in vitro

**Fig. 1:**
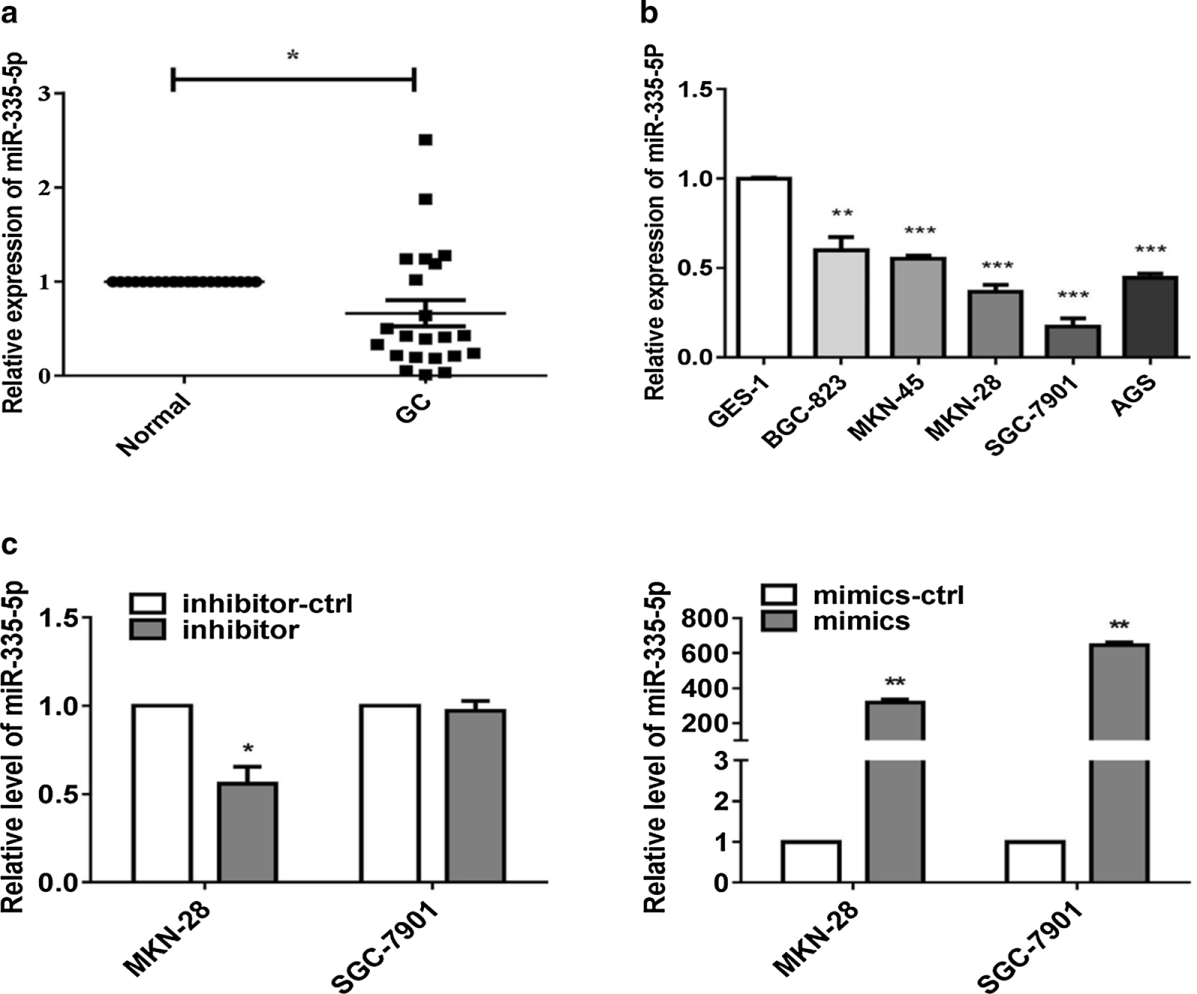
Down-regulation of miR-335-5p in GC tissues and cells. **a** qRT-PCR analysis of miR-335-5p expression in 22 paired human gastric cancer and adjacent normal tissues. The expression of miR-335-5p was normalized to U6. **b** qRT-PCR analysis of miR-335-5p expression in normal gastric mucosal and gastric cancer cells and normalized against U6 RNA. **c** Expression levels of miR-335-5p were determined by qRT-PCR in GCs transfected with miR-335-5p mimics, inhibitor, or respective controls (*P < 0.05, **P < 0.01, and ***P < 0.005)

To investigate the role and function of miR-335-5p in GC cells, we analyzed its expression in 22 pairs of GC tissues and matched adjacent non-cancerous tissue samples by qRT-PCR. miR-335-5p levels were significantly lower in GC samples than in non-cancerous tissue samples (Fig. 1a). These results were validated in five GC cell lines. miR-335-5p levels was lower in the BGC-823, SGC-7901, MKN-45, MKN-28, and AGS cell lines than in the GES-1 cell line (Fig. 1b). To clarify the function of miR-335-5p in GCs, MKN-28 and SGC-7901 cells were selected for further analyses. As determined by qRT-PCR, miR-335-5p mimics successfully elevated miR-335-5p expression in two cell lines; the effect of the inhibitor was moderate due to the low expression of endogenous miR-335-5p in MKN-28 and SGC-7901 cells (Fig. 1c). Thus, miR-335-5p may act as a tumor suppressor in GC.

### miR-335-5p induces cell cycle arrest and apoptosis in GC

**Fig. 2:**
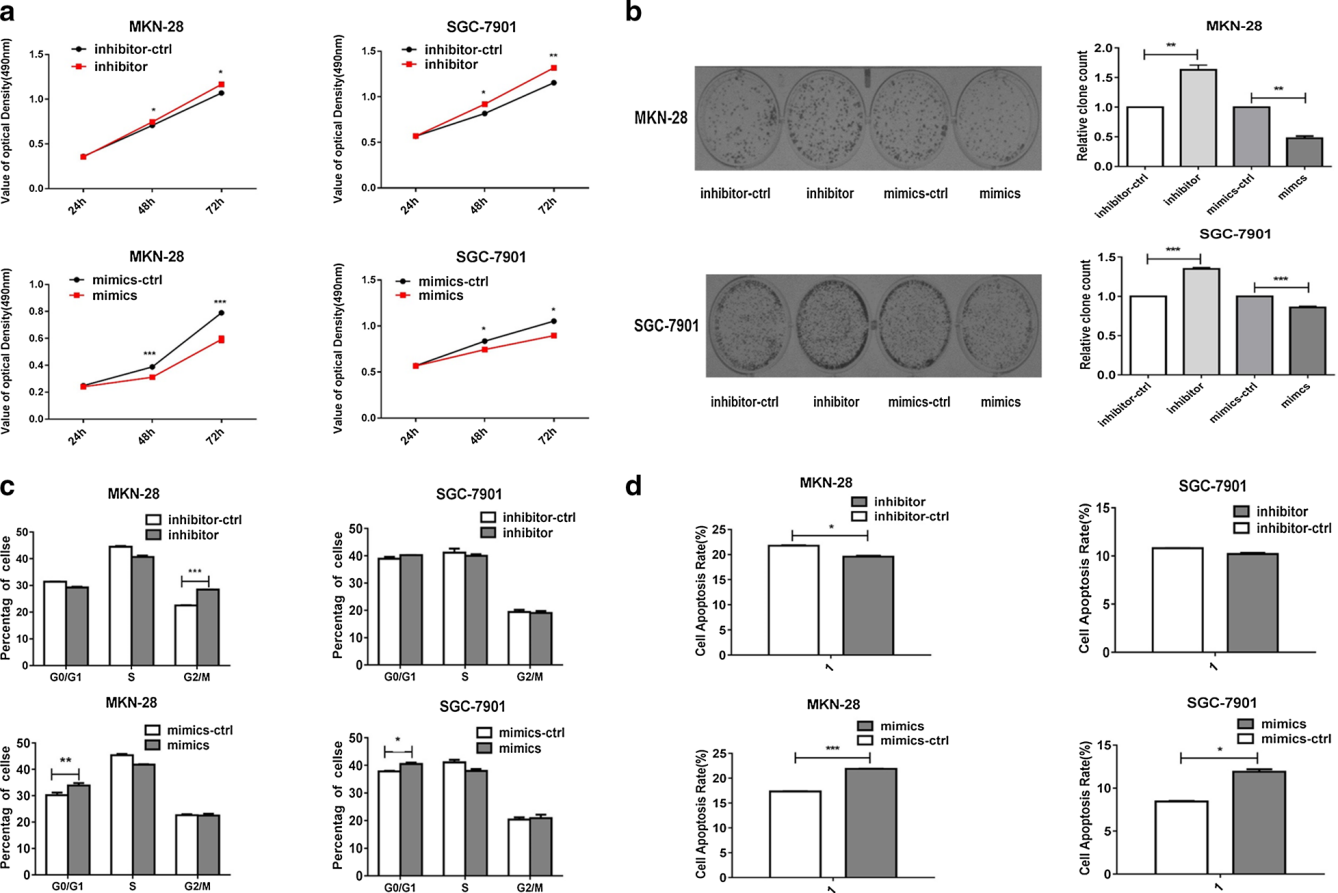
miR-335-5p inhibited proliferation and promoted apoptosis in gastric cancer cells. **a** The effects miR-335-5p on gastric cancer cell proliferation were determined by an MTT assay after the transfection of MKN-28/SGC-7901 cells with an miR-335-5p mimic or miR-335-5p inhibitor at 24, 48, and 72 h. **b** The growth of MKN-28/SGC-7901 cells was detected by colony formation after transfection with the miR-335-5p mimic or inhibitor. **c** Cell cycle progression was evaluated in MKN-28/SGC-7901 cells transfected with miR-335-5p inhibitor-ctrl, inhibitor miR-335-5p ctrl, and mimics. After 48 h, the cell cycle distribution was analyzed by flow cytometry. The histogram indicates the percentages of cells in G0/G1, S, and G2/M phases. **d** Apoptosis was detected by annexin-V/propidium iodide combined labeling flow cytometry in MKN-28/SGC-7901 cells 48 h after transfection with miR-335-5p inhibitor-ctrl, inhibitor, miR-335-5p ctrl, and mimics. Apoptosis was evaluated as the percentage of apoptotic cells (*P < 0.05, **P < 0.01, and ***P < 0.005)

Gain- and loss-of-function analyses were conducted by transfecting MKN-28 and SGC-7901 cells with miR-335-5p inhibitor-ctrl, inhibitor, miR-ctrl, and mimics. MTT and colony formation assays showed that the upregulation of miR-335-5p in MKN-28 and SGC-7901 cells inhibited cell growth and colony formation, while the inhibition of miR-335-5p exerted moderate adverse effects on GC cells, which may be explained by the low levels of miR-335-5p in MKN-28 and SGC-7901 cells (Fig. 2a, b). Consistent with these results, a flow cytometry analysis revealed that the upregulation of miR-335-5p arrested cells in the G0/G1 phase and inhibited the transition to the G2/M phase; similar effects were not observed in the miR-ctrl-transfected cells (Fig. 2c). Furthermore, flow cytometry confirmed that the upregulation of miR-335-5p induces apoptosis in GC cells. However, the miR-335-5p inhibitor resulted in a slight but non-significant difference in apoptosis compared to that in cells transfected with the negative control, which may be explained by the low expression level and low inhibitory efficiency in MKN-28 and SGC-7901 cells (Fig. 2d). The inhibition of MiR-335-5p promoted proliferation and inhibited apoptosis in GC cells, while the inverse results were obtained in the miR-335-5p mimic group.

### Inhibition of miR-335-5p induces the migration and invasion of gastric cancer cells

**Fig. 3:**
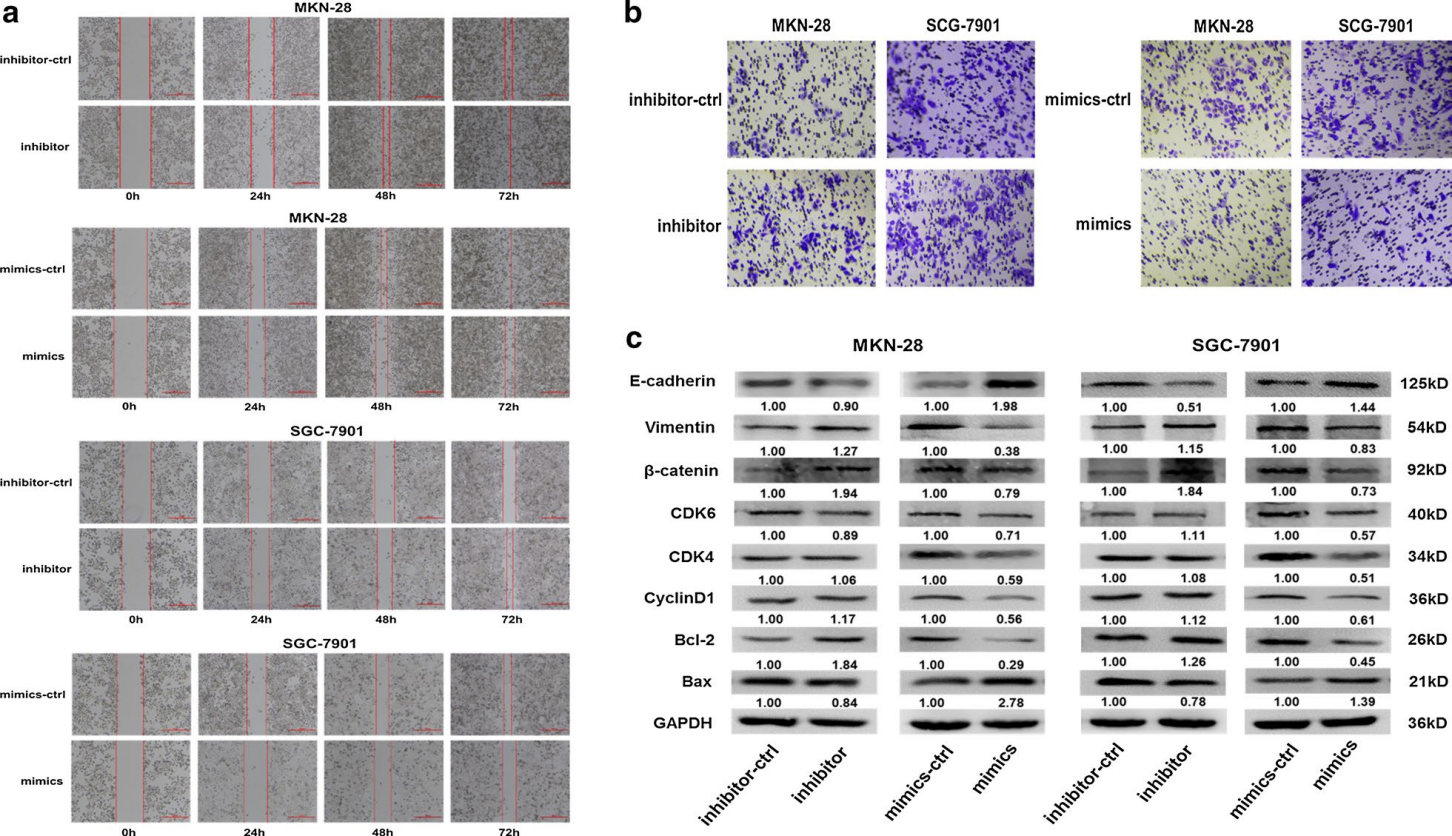
miR-335-5p inhibited the migration and invasion of MKN-28 and SGC-7901 cells. **a** Scratch wound-healing assays of MKN-28 and SGC-7901 cells after treatment with miR-335-5p inhibitor-ctrl, inhibitor, miR-ctrl, or miR-335-5p mimics. **b** Transwell analysis of MKN-28 and SGC-7901 cells after transfection with miR-335-5p mimics, inhibitor, or their respective controls. **c** Western blot analysis of CDK6, CDK4, CyclinD1, BCL-2, BAX, E-cadherin, Vimentin, and β-catenin expression in MKN-28 and SGC-7901 cells transfected with miR-335-5p, inhibitor, or their respective controls

To further confirm that miR-335-5p acts as a tumor suppressor, its effects on the invasion of MKN-28 and SGC-7901 cells were evaluated by a Transwell invasion assay and wound-healing assay. In the wound-healing assay, migration was slower in the miR-335-5p-transfected cells than in un-transfected cells. Over time, the difference in the metastasis rate between the two groups increased (Fig. 3a). In the Transwell invasion assay, the transfection of cells with miR-335-5p mimics significantly impaired invasion compared to that in the miR-335-5p-ctrl group in MKN-28 and SGC-7901 cells. In contrast, the knockdown of miR-335-5p enhanced GC cell invasion. When transfected with the mir-335-5p inhibitor, the MKN-28 and SGC-7901 cell invasion rates increased significantly (Fig. 3b). These results support the hypothesis that miR-335-5p contributes to the suppression of invasion and metastasis. To investigate the mechanisms underlying the roles of miR-335-5p in apoptosis and cell cycle progression, we measured the expression levels of apoptosis- and cell cycle-related proteins in GC cells. The transfection of MKN-28/SGC-7901 cells with MiR-335 downregulated CDK6, CDK4, CyclinD1, and BCL-2 and upregulated the expression of BAX. The overexpression of miR-335-5p reduced the expression levels of vimentin and β-catenin, and significantly increased E-cadherin levels in MKN-28 and SGC-7901 cells. Our results showed that the silencing of miR-355-5p significantly increased the relative expression levels of vimentin and β-catenin and decreased E-cadherin expression, comparable with the effects of miR-355-5p overexpression in MKN-28 and SGC-7901 cells (Fig. 3c). These results suggest that mir-335-5p is involved in the progression, migration, and invasion of GCs.

### MAPK10 is a direct functional target of miR-335-5p in GC cells

**Fig. 4:**
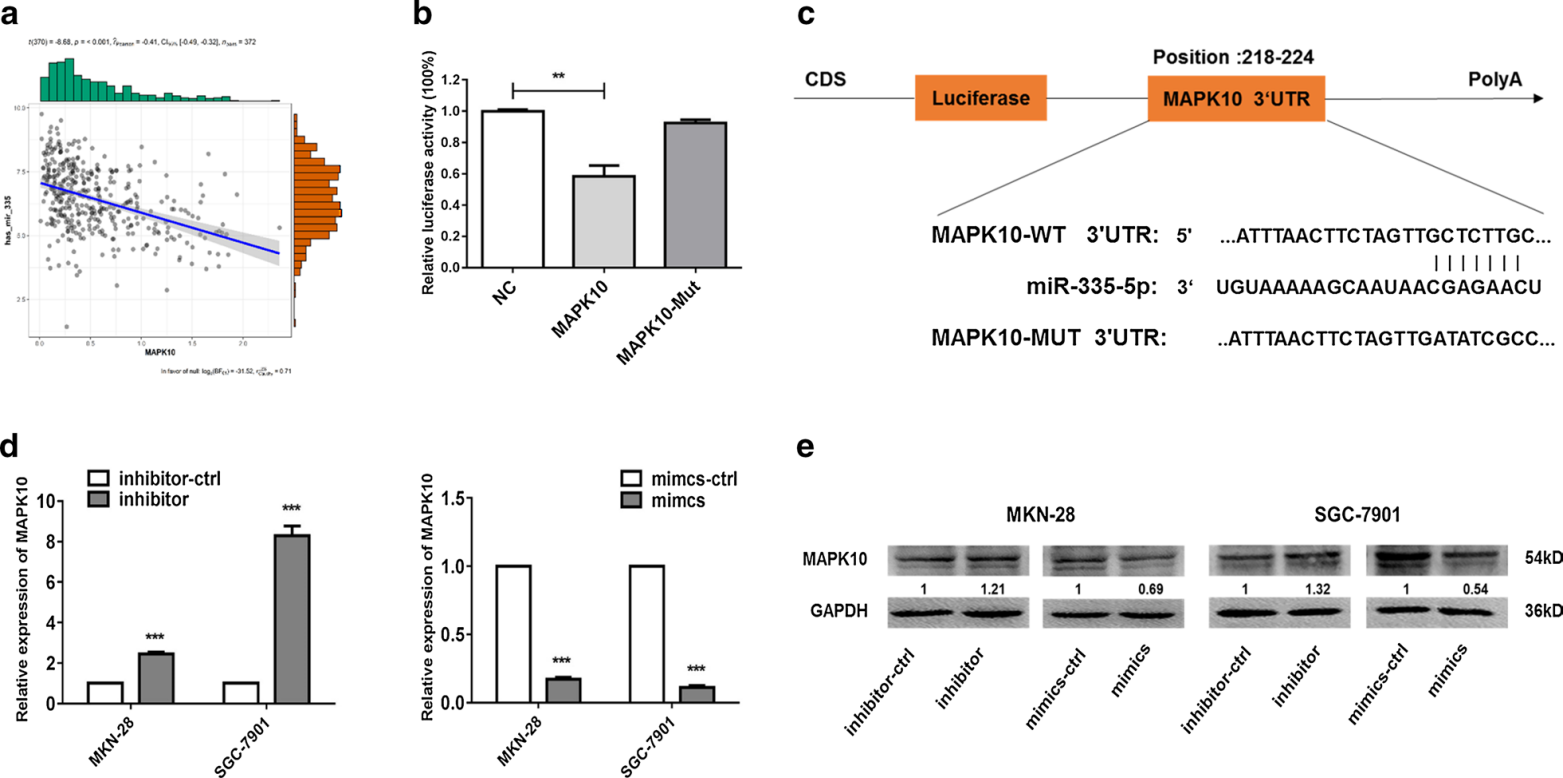
MAPK10 is a direct target of miR-335-5p in gastric cancer cell lines. **a** Correlation between MAPK10 expression and miR-335-5p expression in gastric cancer based on TCGA data. **b** A luciferase assay was performed using HEK293 cells in which miR-335 was co-transfected with the pGLO-MAPK10 wild-type or pGLO-MAPK10 mutant vector. **c** miR-335-5p is highly conserved across species and has binding sites within the 3′-UTR of human MAPK10. **d** mRNA expression levels of MAPK10 were measured by qRT-PCR after transfection with miR-335-5p mimics, miR-335-5p inhibitor, or their negative controls in MKN-28 and SGC-7901 cells. **e** Protein expression levels of MAPK10 were measured by western blotting after transfection with miR-335-5p mimics, miR-335-5p inhibitor, or their respectively negative controls in MKN-28 and SGC-7901 cells (*P < 0.05, **P < 0.01, and ***P < 0.005)

miRNA target prediction algorithms were used to search for potential miR-335-5p target genes. Levels of MAPK10 and mir-335-5p expression in GC based on TCGA data showed a negative correlation (*P* < 0.001; Fig. 4a). MAPK10 had a potential miR-335-5p-binding site in the 3′-UTR and therefore was selected as a candidate target. To determine whether MAPK10 was directly targeted by miR-335-5p, we subcloned 3′-UTR MAPK10 fragments including wild-type (MAPK10-WT) and mutant (MAPK10-MUT) miR-335-5p-binding sites into the pmiRGLO dual-luciferase reporter vector (Fig. 4b). Pre-miR-335 and MAPK10-WT- or MUT-3′-UTR vectors were co-transfected into HEK293 cells. The relative luciferase activity of the MAPK10-WT pmirGLO-3-UTR vector was significantly reduced in miR-335-overexpressing HEK293 cells. As expected, miR-335-5p failed to inhibit the luciferase activity of the MAPK10-MUT pmirGLO-3′-UTR vector, indicating that miR-335-5p binds directly to the 3′-UTR of MAPK10 (Fig. 4c). qRT-PCR was used to verify the relationship between miR-335-5p and MAPK10. The mRNA levels of MAPK10 decreased significantly by miR-335 mimics and increased by miR-335 inhibitors in MKN-28 and SGC-7901 cells (Fig. 4d). The protein levels of MAPK10 decreased significantly by miR-335 mimics and increased by miR-335 inhibitors in MKN-28 and SGC-7901 cells (Fig. 4e). These findings demonstrated that miR-335-5p could directly target MAPK10 and suppress its expression in GC cells.

### Bioinformatics analysis of MAPK10 in gastric cancer

**Fig. 5:**
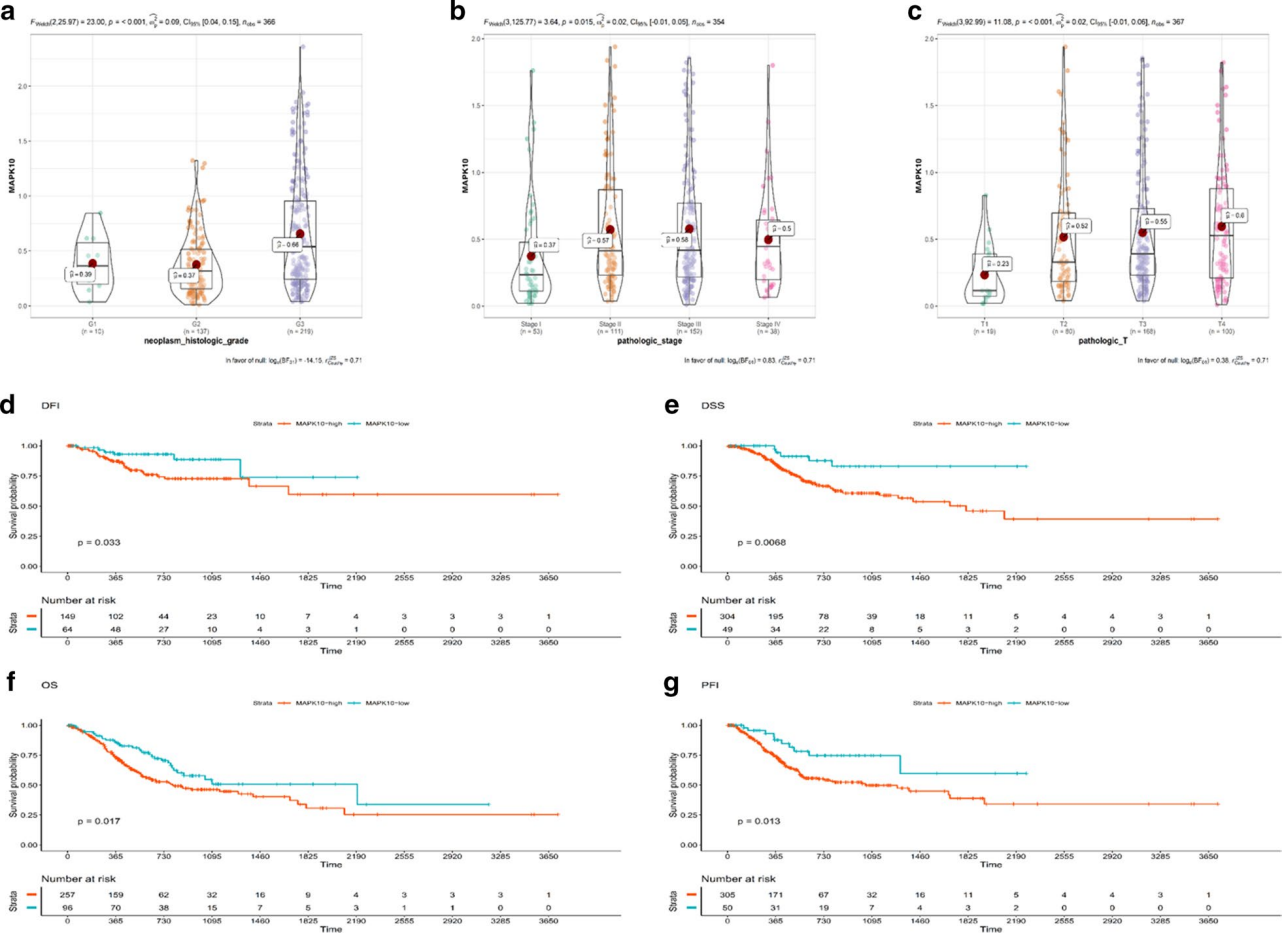
**a**–**c** The expression of MAPK10 is associated with the pathologic and histologic grade of GC. **d**–**g** Bioinformatics analyses were used to elucidate the effect of MAPK10 in GC tissues. The expression of MAPK10 was related to the DFI (disease-free interval event), PFI (progression-free interval event), DSS (disease-specific survival event), and OS (overall survival)

The TCGA database was used to elucidate the effect of MAPK10 in GC tissues by a bioinformatics approach. The expression of MAPK10 was higher in GC tissues than in healthy counterparts, and its expression was associated with the histologic and pathologic stages of GC (Fig. 5a–c). The expression of MAPK10 was associated with the DFI (disease-free interval, *P* = 0.033), PFI (progression-free interval, *P* = 0.013), DSS (disease-specific survival, *P* = 0.0068), and OS (overall survival, *P* = 0.017) in GC (Fig. 5d–g), suggesting that MAPK10 plays a key role as an oncogene in GC.

### Knockdown of MAPK10 reduces GC progression

**Fig. 6:**
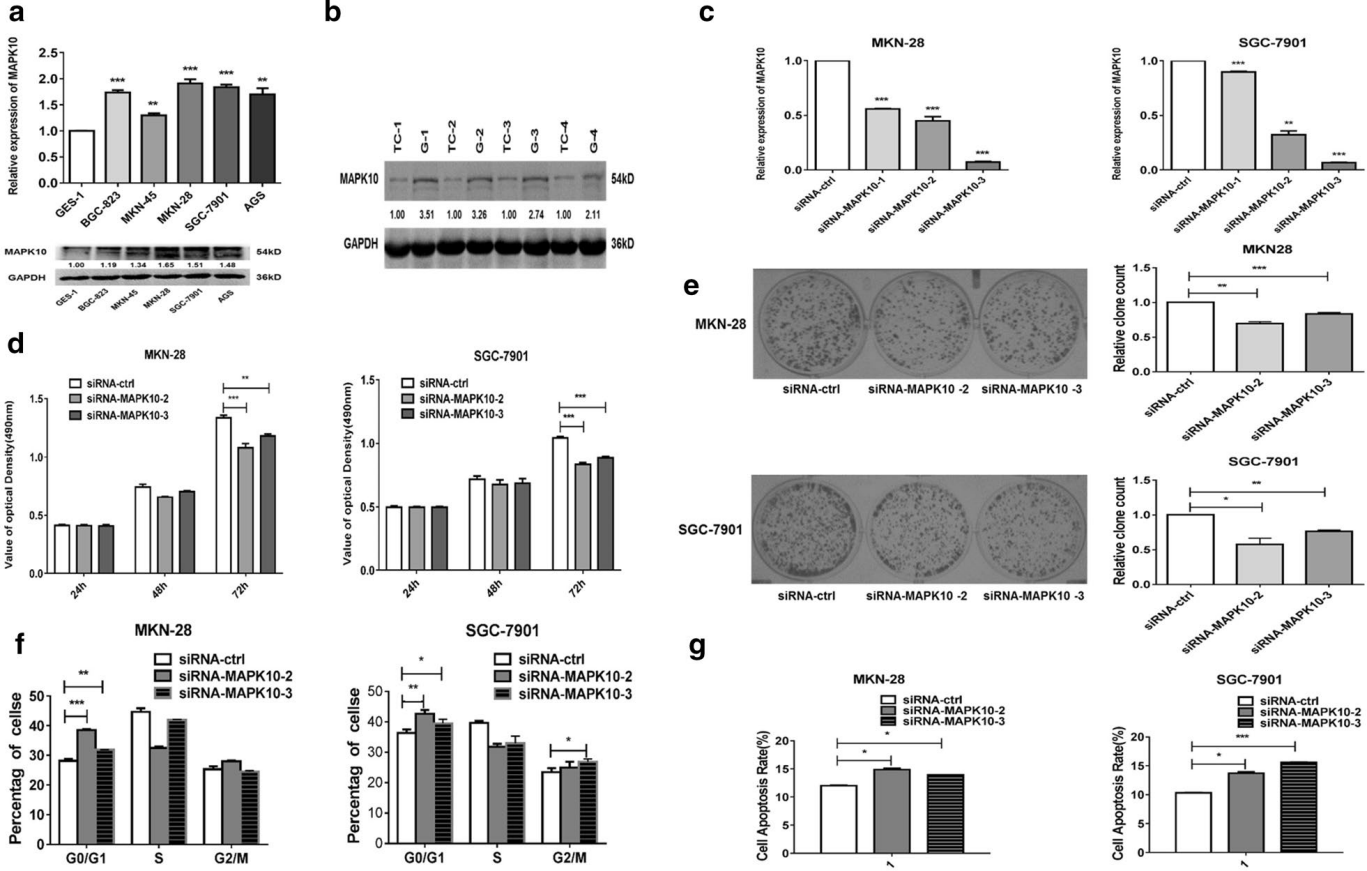
Inhibition of MAPK10 suppressed GC progression. **a** mRNA and protein expression levels of MAPK10 in various GC and GES-1 cells. **b** MAPK10 protein expression in GC tissues vs counterparts' tissues was confirmed by using western blotting. **c** Expression levels of MAPK10 were measured by qRT-PCR in MKN-28 and SGC-7901 cells transfected with siMAPK10. **d** An MTT assay was performed to determine the growth of gastric cancer cells treated with siMAPK10 or a negative control (si-ctrl). **e** A colony formation assay was performed several days after the transfection of gastric cancer cells with siMAPK10 or a negative control (si-ctrl). **f** The cell cycle distribution was determined in gastric cancer cells 48 h after transfection with siMAPK10 by propidium iodide staining and flow cytometry. The histogram indicates the percentages of cells in G0/G1, S, and G2/M cell cycle phases. **g** Apoptosis was determined in gastric cancer cells at 48 h after transfection with siMAPK10 (*P < 0.05, **P < 0.01, and ***P < 0.005)

We knocked down MAPK10 expression by RNA interference [small interfering RNA (siRNA)] to confirm that MAPK10 mediates the antitumor effects of miR-335-5p. MAPK10 expression levels were higher in GC cells than in GES-1 cells (Fig. 6a) and were highest in MKN-28 and SGC-7901 cells. Western blotting indicated that the MAPK10 was obviously up-regulated in GC tissues than in their counterparts at the protein level (Fig. 6b). MAPK10 was successfully knocked down by siRNA, as verified by analyses of both at the mRNA levels (Fig. 6c). Similar to miR-335-5p-overexpressing cells, the downregulation of MAPK10 significantly inhibited proliferation and slightly inhibited colony formation in MKN-28 and SGC-7901 cells (Fig. 6d, e). Moreover, the influence of MAPK10 siRNA on the cell cycle was similar to the effect of miR-335-5p upregulation (Fig. 6f). Consistent with the effect of miR-335-5p on GC cell apoptosis, MAPK10 knockdown induced apoptosis in MKN28/SGC-7901 cells (Fig. 6g), suggesting that MAPK10 is involved in the progression of GC.

### Knockdown of MAPK10 reduces the migration and invasion of GC cells

**Fig. 7:**
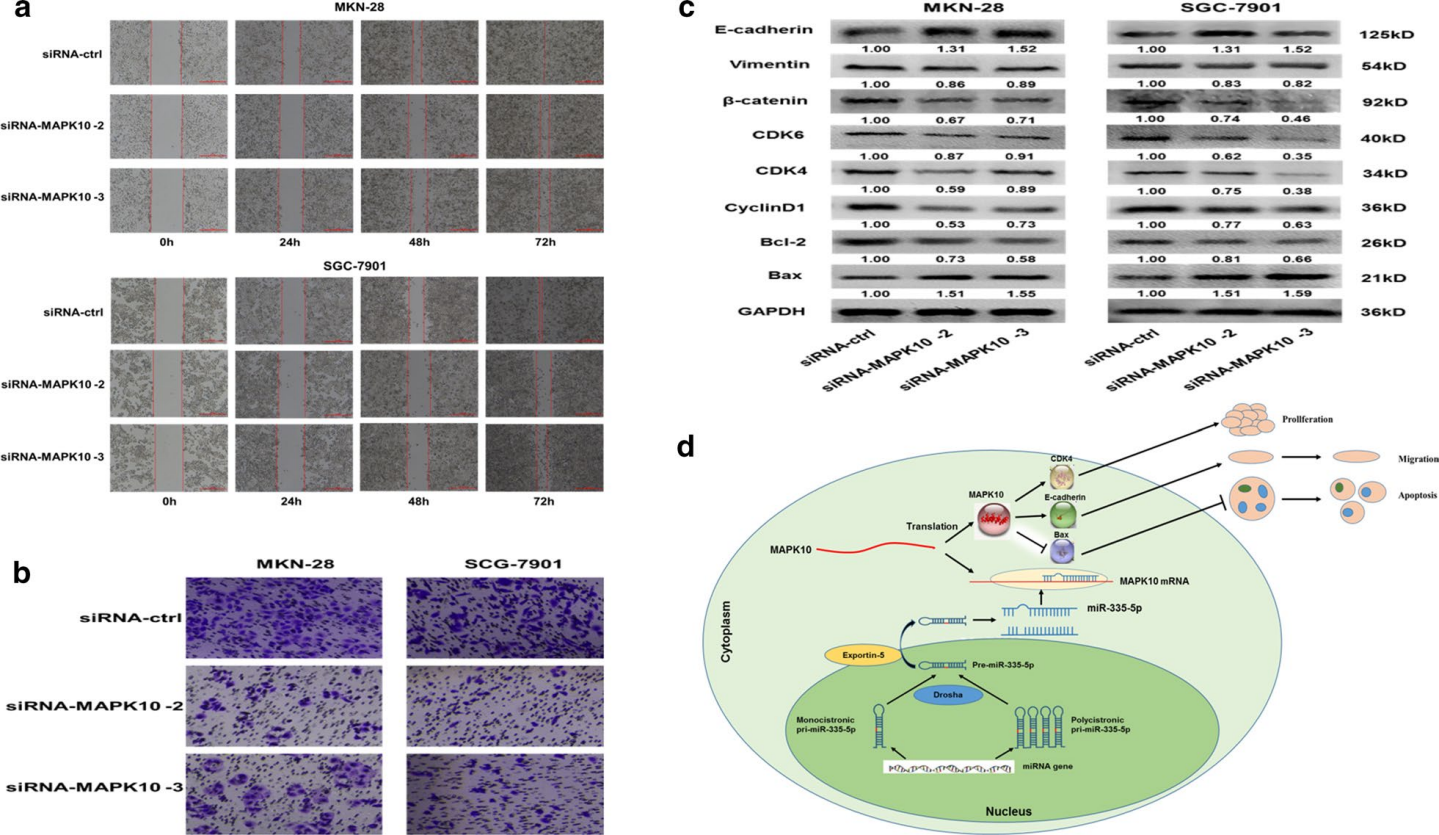
miR-335-5p inhibited cell invasion via MAPK10 knockdown. **a** Wound-healing assays of MKN-28 and SGC-7901 cells after treatment with si-ctrl and si-MAPK10. Representative images were captured at 0 h, 24 h, 48 h, and 72 h after transfection with si-ctrl and si-MAPK10. **b** The invasion viability of MKN-28 and SGC-7901 cells transfected with siMAPK10 was determined by a Transwell invasion assay. **c** The expression levels of MAPK10 were measured by western blotting in MKN-28/SGC-7901 cells transfected with siMAPK10. Protein expression levels of CDK6, CDK4, CyclinD1, BCL-2, BAX, E-cadherin, Vimentin, and β-catenin in gastric cancer cells transfected with siMAPK10 or si-ctrl were analyzed by western blotting. **d** Proposed model for the suppressive effect of miR-335-5p on gastric cancer progression via MAPK10 knockdown

We silenced MAPK10 expression by RNA interference (RNAi) to evaluate whether it contributes to the effects of miR-335-5p on invasion and metastasis using MKN-28 and SGC-7901 cells. Based on a wound-healing assay, the group with low MAPK10 expression showed reduced rates of migration (Fig. 7a). Transwell assays demonstrated that MAPK10 silencing inhibited the invasion and migration ability of GC cells (Fig. 7b). Based on a western blot analysis, silencing MAPK10 significantly increased the relative expression levels of E-cadherin and decreased vimentin and β-catenin expression. These results were consistent with the effects of miR-355-5p overexpression in MKN-28 and SGC-7901 cells (Fig. 7c), suggesting that MAPK10 functions as an oncogene in GC. We concluded that miR-335-5p suppresses GC progression by targeting MAPK10 (Fig. 7d).

## Discussion

The occurrence of GC is a complex process involving multiple factors, including genetic and epigenetic events. Many miRNAs have been demonstrated to contribute to GC tumorigenesis and development, and these serve as a valid diagnostic and therapeutic targets [28]. In addition, some specific microRNAs able to regulate the expression of genes in GC cells at the post-transcriptional level have been identified as diagnostic biomarkers for GC [29–31]. Therefore, studies of potential miRNAs associated with the development of GC may provide opportunities for improvements in diagnosis, treatment, and prognosis.

In this study, the roles of mir-335-5p in GC were evaluated. Recent studies have shown that miR-335-5p acts as a tumor suppressor and inducer in several cancer types [32–35]. For example, miR-335-5p is downregulated in thyroid cancer cells and inhibits proliferation [36]. miR-335-5p functions via lactate dehydrogenase B to exert tumor inhibitory effects in colorectal cancer [37]. In the present study, based on expression analyses of miR-335-5p in tissues, we found that miR-335-5p acts as a tumor suppressor in GC, and the overexpression of miR-335-5p inhibits proliferation, invasion, and metastasis and induces apoptosis in vitro. Additionally, miR-335-5p could induce cell cycle arrest at the G1 phase and downregulated the G0 and G1 phase cycle-associated proteins Cyclin D1, CDK 6, and CDK4. The abnormal activation of CDK and its modulators has been reported in many tumors [38, 39]. Furthermore, miRNAs participate in the regulation of the cell cycle [40, 41]. For example, the miR-15a/16 family regulates G0/G1 cell cycle progression by targeting cyclin D1 (CCND1) [42] addition, miR-16 regulates various mRNA targets, including CDK6, CDC27, and G1-related cyclins, which jointly control cell cycle progression [43]. These studies strongly support our observation that miR-335-5p plays a key role in cell proliferation in GC.

Migration and invasion are closely related to the occurrence and development of tumors. Moreover, many migration- and invasion-related proteins, including E-cadherin, vimentin, and β-catenin, are involved. For instance, p0071 interacts with E-cadherin in the cytoplasm and promotes invasion and metastasis in non-small cell lung cancer [44]. Furthermore, ubiquitin specific peptidase 20 (USP20) regulates the deubiquitination of β-catenin to control the invasion and migration of cancer cells [45]. Our results showed that the overexpression of mir-335-5p decreases the expression of vimentin and β-catenin and increases E-cadherin in MKN-28 and SGC-7901 cells. Our data suggest that mir-335-5p is involved in the migration and invasion of GCs.

In addition, our results showed that the upregulation of miR-335-5p inhibits the expression of MAPK10 in MKN-28/SGC-7901 cancer cells at the RNA and protein levels. Using bioinformatic analyses and a dual-luciferase reporter assay, we demonstrated that miR-335-5p directly targets MAPK10 by binding to its 3′-UTR and inhibiting translation. To further clarify the tumor suppressive effect of miR-335-5p via MAPK10, siRNA was used. MAPK10 silencing inhibited cell proliferation and migration and induced cell apoptosis, similar to the observed effects of miR-335-5p overexpression in GC cells in vitro. Accordingly, the expression levels of related proteins, including CDK6, CDK4, CyclinD1, BCL-2, BAX, E-cadherin, vimentin, and β-catenin were also altered by siMAPK10. MAPK10 is a member of the Jun N-terminal kinase subgroup of the mitogen-activated protein kinases, which are implicated in important physiological processes [46]. MAPK10 regulates the occurrence and development of several types of cancer. The downregulation of MAPK10 contributes to the suppression of ovarian cancer [47]. miR-27a-3p promotes the growth and invasion of NPC cells by targeting Mapk10 [48]. These results robustly suggested that the downregulation of MAPK10 induced by miR-335-5p could inhibit GC progression. Our findings highlight that mir-335-5p or MAPK10 may be considered as potential targets for GC therapy in the near future.

## Conclusion

We obtained the following new findings. (1) miR-335-5p functions as a tumor suppressor in GC. (2) miR-335-5p leads to the inhibition of proliferation and metastasis and the promotion of apoptosis in GC cells. (3) MAPK10 is a downstream target gene of miR-335-5p. (4) MAPK10 has a vital role as an oncogene in GC.

## Data Availability

The datasets supporting the conclusions of this study are included in this article. Any requests for data or materials can be sent to the corresponding author.

## Abbreviations

miRNAs: MicroRNAs
GC: Gastric cancer
MAPK10: Mitogen‑activated protein kinase 10
GAPDH: Glyceraldehyde-3-phosphate dehydrogenase
TBST: Tris buffer solution with tween
siRNA: Small interfering RNA
